# Beyond BRUE (Brief Resolved Unexplained Event): Recurrent Unexplained Events Revealing Congenital Hyperinsulinism in Infancy

**DOI:** 10.7759/cureus.108028

**Published:** 2026-04-30

**Authors:** Mudassir A Khan, Fasih khan, Nimra Chohan, Shatha Alsamawi

**Affiliations:** 1 Pediatrics, Marshall University Joan C. Edwards School of Medicine, Huntington, USA; 2 Pediatrics, Aga Khan University Hospital, Karachi, PAK

**Keywords:** brue, congenital hyperinsulinism, diazoxide-responsive hypoglycemia, glucagon stimulation test, hyperinsulinemic hypoglycemia

## Abstract

Brief resolved unexplained events (BRUEs) are uncommon yet clinically significant presentations in infancy that require careful risk stratification, as recurrent episodes may herald serious occult pathology. We report a four-month-old term female infant with a notable family history of sudden unexplained sibling deaths who was admitted to and later discharged from our institution twice for recurrent BRUE episodes following an initially unrevealing workup. She subsequently presented again with vomiting, diarrhea, dehydration, metabolic acidosis, and persistent hypoglycemia, necessitating admission to the pediatric intensive care unit (PICU).

During PICU admission, the infant developed recurrent hypoglycemia despite escalating intravenous glucose infusion rates (GIRs). Critical sampling demonstrated inappropriately detectable insulin and C-peptide, absent ketonuria, suppressed β-hydroxybutyrate, and a diagnostically significant glycemic response to glucagon, confirming hyperinsulinemic hypoglycemia (HH) consistent with congenital hyperinsulinism (CHI). She responded promptly to diazoxide and hydrochlorothiazide, allowing successful weaning from intravenous glucose and transition to full oral feeds. This case compellingly demonstrates how seemingly benign recurrent BRUE episodes may, in fact, constitute the earliest clinical clue to a serious yet treatable occult disorder.

## Introduction

In 2016, the American Academy of Pediatrics (AAP) introduced the term brief resolved unexplained event (BRUE) to replace the earlier designation of apparent life-threatening event (ALTE). BRUE refers to a sudden, brief, and completely resolved episode in an infant younger than one year characterized by cyanosis or pallor; absent, decreased, or irregular breathing; marked change in tone; or altered responsiveness, with no explanation identified after an appropriate history and physical examination [1]. Reported incidence rates are approximately 4.28 per 1,000 live births and 5.06 per 1,000 infant emergency department encounters [2]. Although most BRUE episodes are ultimately benign, they may occasionally represent the sentinel presentation of significant underlying disease, necessitating a thorough and systematic diagnostic approach.

Hypoglycemia in infants can closely resemble BRUE, with overlapping features such as pallor, cyanosis, apnea, hypotonia, poor feeding, lethargy, irritability, altered responsiveness, and even seizures. A key cause of persistent hypoglycemia in this age group is congenital hyperinsulinism (CHI), characterized by inappropriate insulin secretion despite low plasma glucose levels. The estimated incidence of CHI is approximately one in 50,000 live births, rising to as high as one in 2,500 in populations where consanguinity is more prevalent [3]. Given this overlap, infants with recurrent BRUE-like episodes should be carefully evaluated for underlying metabolic or endocrine etiologies, including CHI. We describe a case in which recurrent BRUE presentations led to the identification of hypoglycemia and ultimately the diagnosis of CHI. 

## Case presentation

We report the case of a four-month-old female infant admitted to the pediatric intensive care unit (PICU) with hypoglycemia, metabolic acidosis, and dehydration in the setting of decreased oral intake, persistent vomiting, and diarrhea for two days. She was born at term and appropriate for gestational age via elective cesarean section following a pregnancy without significant complications. The mother had a remote history of substance use disorder but had been in sustained remission for two years prior to delivery. The infant’s immediate neonatal course was unremarkable, with Apgar scores of 9 at both one and five minutes and no documented neonatal hypoglycemia, and she was discharged home from the newborn nursery in stable condition. 

The family history was notable for the sudden, unexplained deaths of two half-siblings aged five and four years. Although the exact causes were unknown, this history raised concern for a potential inherited cardiac or metabolic disorder; therefore, the infant was kept under close clinical surveillance with frequent follow-up visits. In the months following discharge from the newborn nursery, the infant was admitted twice for BRUEs. Each episode involved choking followed by breath-holding, with rapid and complete return to baseline. During the first admission, evaluation included electrocardiography, a viral respiratory panel, and overnight cardiorespiratory monitoring, all of which were unremarkable. No laboratory investigations or glucose measurements were obtained, and the event was attributed to possible gastroesophageal reflux in the context of a recent formula change. 

**Figure 1:**
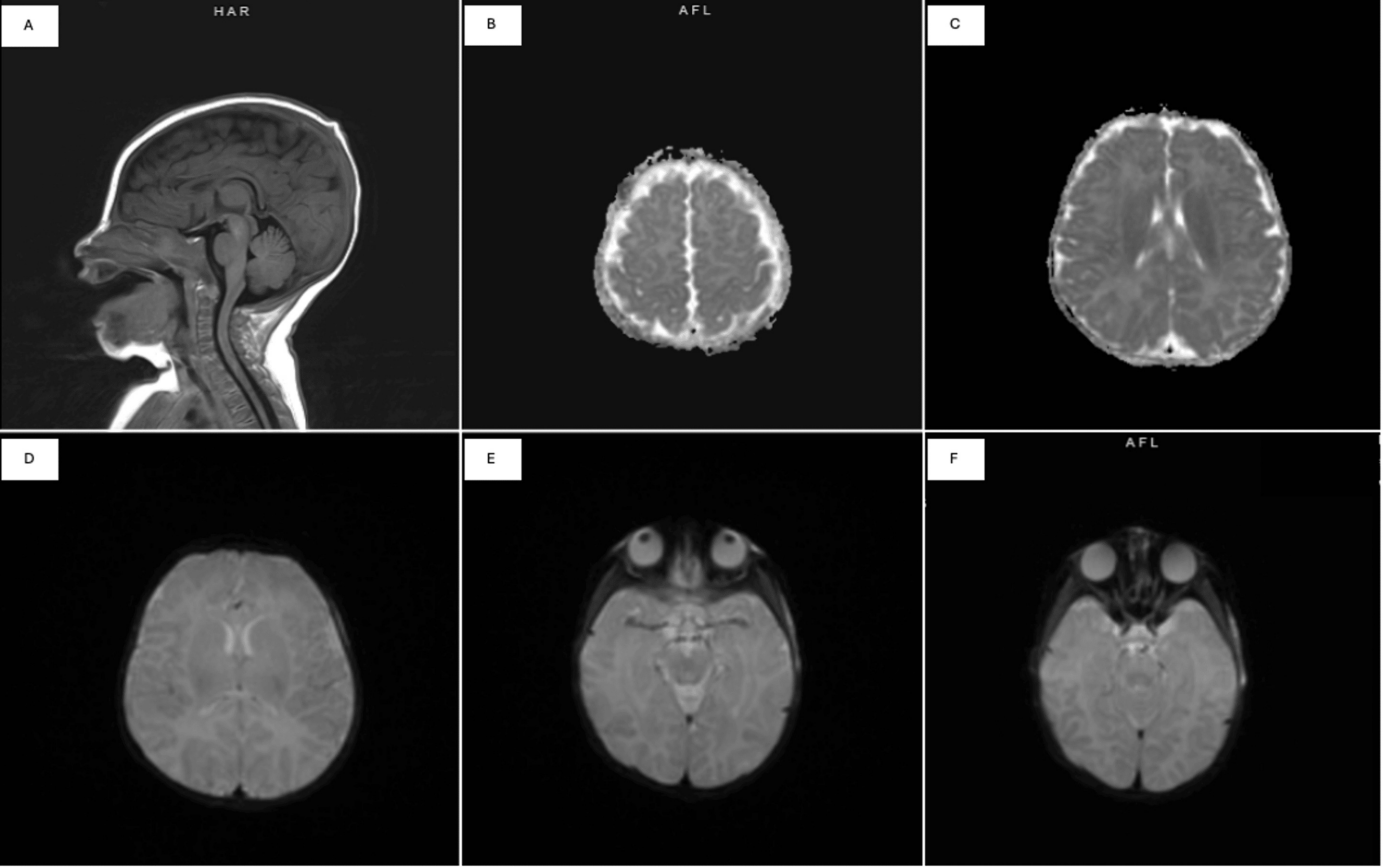
MRI brain without contrast MRI: magnetic resonance imaging; DWI: diffusion-weighted imaging (A) Sagittal T1-weighted image demonstrating normal brain anatomy without structural abnormalities. (B-C) Axial T2-weighted images showing symmetric cerebral hemispheres without focal lesions. (D) DWI without evidence of restricted diffusion. (E-F) Additional axial sequences demonstrating no evidence of hypoxic-ischemic injury or intracranial pathology

Following a second admission within a week, a more comprehensive evaluation was performed. Echocardiography revealed a small secundum atrial septal defect with left-to-right shunting. Notably, serum glucose levels were assessed during this admission and remained within normal limits. Additional studies, including electrocardiography, abdominal ultrasound, and brain MRI, were unremarkable. The infant was subsequently discharged on famotidine and an extensively hydrolyzed formula for presumed gastroesophageal reflux disease (GERD). Figure 1 illustrates the MRI brain without contrast.

Two weeks prior to presentation, the infant experienced an episode characterized by fixed staring, upward eye deviation, and generalized tonic movements lasting less than one minute. She was evaluated in the emergency department, observed for six hours without recurrence, and discharged home without further evaluation; no lab investigation, glucose monitoring or EEG was performed. The mother also reported at least two prior brief similar episodes that had not been medically assessed. 

Two days prior to current admission, the infant was reportedly in her usual state of health when she developed increased spit-ups followed by recurrent postprandial vomiting. The emesis was nonbloody, nonbilious, and nonprojectile. She also had four episodes of foul-smelling, nonbloody diarrhea. These symptoms were accompanied by decreased oral intake, reduced urine output with fewer wet diapers, and diminished activity compared to baseline, prompting presentation to the emergency department. The mother denied fever, cough, congestion, or rash. The clinical timeline of events is shown in Table 1. 

**Table 1:** Clinical timeline of events PICU: pediatric intensive care unit; BRUE: brief resolved unexplained event; ED: emergency department; EEG: electroencephalogram; GERD: gastroesophageal reflux disease; CRM: cardiac rhythm management

Age/time point	Clinical event	Key findings/management
Birth	Term female infant born via elective cesarean section, appropriate for gestational age	No neonatal hypoglycemia. Discharged from a newborn nursery in stable condition
First hospitalization (2 months old)	Admission for BRUE	Normal EKG and overnight CRM. No laboratory testing or glucose monitoring were performed
Second hospitalization (within 1 week of discharge)	Recurrent BRUE	Detailed workup negative. Normal Serum glucose levels. Discharged on famotidine and an extensively hydrolyzed formula for possible GERD
2 weeks before the current admission	ED visit for an episode concerning a seizure	Evaluated in ED, observed for 6 hours without recurrence, and discharged home. No lab investigation, glucose monitoring, or EEG performed
Current ED visit and admission to PICU (4 months old)	Dehydration, hypoglycemia, and metabolic abnormalities	Received IV fluids and IV dextrose bolus and was later admitted

On arrival at the emergency department, the infant appeared clinically dehydrated and received an intravenous fluid bolus and ondansetron. Initial laboratory evaluation demonstrated metabolic acidosis (serum bicarbonate 16 mmol/L) and hypoglycemia, with a point-of-care glucose of 60 mg/dL, improving to 65 mg/dL after an intravenous dextrose bolus. The comprehensive metabolic panel revealed transaminitis (aspartate aminotransferase (AST): 131 U/L; alanine aminotransferase (ALT): 226 U/L), which could be explained in the setting of dehydration due to ongoing gastrointestinal losses. Complete blood count showed persistent neutropenia with an absolute neutrophil count of 0.93 K/µL. Urinalysis was notable only for trace ketones (5 mg/dL), while the viral respiratory panel and inflammatory markers were negative. Initial lab workup upon admission is shown in Tables 2-3. 

**Table 2:** Complete blood count WBC: white blood cell; MCV: mean corpuscular volume; ANC: absolute neutrophil count

Parameter	Patient value	Normal range
WBC	7.13 K/µL	5.0-15.0 K/µL
Hemoglobin	11.20 g/dL	10.50-13.50 g/dL
Hematocrit	30.50%	33-39%
Platelet count	269 K/µL	150-450 K/µL
MCV	79 fL	70-86 fL
Segmented neutrophils	6%	15-45%
Lymphocytes	65%	45-75%
ANC	0.93 K/µL	1.0-8.50 K/µL

**Table 3:** CMP, inflammatory markers, and viral panel CMP: comprehensive metabolic panel; AST: aspartate aminotransferase; ALT: alanine aminotransferase; CRP: C-reactive protein

Parameter	Patient value	Normal range
Sodium	136 mmol/L	135-145 mmol/L
Potassium	4.90 mmol/L	3.50-5.0 mmol/L
Chloride	108 mmol/L	98-107 mmol/L
Bicarbonate	16 mmol/L	22-28 mmol/L
BUN	10 mg/dL	5-18 mg/dL
Creatinine	0.20 mg/dL	0.20-0.40 mg/dL
Glucose	69 mg/dL	70-110 mg/dL
Calcium	9.60 mg/dL	8.50-10.50 mg/dL
Total protein	5.60 g/dL	6.0-8.0 g/dL
Albumin	4.20 g/dL	3.50-5.0 g/dL
Alkaline phosphatase	176 U/L	150-420 U/L
AST	131 U/L	20-60 U/L
ALT	226 U/L	12-45 U/L
Total bilirubin	0.30 mg/dL	0.20-1.0 mg/dL
CRP	0.60 mg/dL	<1.0 mg/dL
Procalcitonin	0.08 ng/mL	<0.50 ng/mL
Viral panel	Negative	Negative

Upon admission to the PICU, the infant was initiated on intravenous maintenance fluids consisting of D10W in 0.9% sodium chloride, delivered at a maintenance rate with a calculated glucose infusion rate (GIR) of 6.6 mg/kg/min. Point-of-care glucose monitoring was performed hourly, and continuous neurological observation was instituted to monitor for potential seizures precipitated by hypoglycemia. Given the elevated transaminases, an abdominal ultrasound was obtained, and a comprehensive infectious workup, including Epstein-Barr virus (EBV), cytomegalovirus (CMV), and viral hepatitis panel, was performed, all of which were within normal limits. 

Pediatric endocrinology and genetics services were consulted early in the admission to evaluate for possible inborn errors of metabolism and endocrine causes of hypoglycemia. The consulting teams recommended obtaining a comprehensive critical sample during any hypoglycemic episode. Suggested studies included plasma amino acids, urinary organic acids, an acylcarnitine profile, along with serum insulin, C-peptide, β-hydroxybutyrate, growth hormone, cortisol, lactate, uric acid, lipid panel, ammonia, pyruvate, and urine ketones. The interpretation of the above given lab workup and its possible associated pathologies are reported in Table 4. 

**Table 4:** Hypoglycemia critical sample: interpretation of laboratory tests PRPP: phosphoribosyl pyrophosphate; MCAD: medium-chain acyl-CoA dehydrogenase; VLCAD: very long-chain acyl-CoA dehydrogenase; CPT II: carnitine palmitoyltransferase II

Test	What it evaluates	Example disorders
Plasma amino acids	Screening for amino-acidopathies and some organic acidemias	Maple syrup urine disease, phenylketonuria, homocystinuria
Urinary organic acids	Detection of organic acidemias	Methylmalonic acidemia, propionic acidemia, isovaleric acidemia
Acylcarnitine profile	Fatty acid oxidation defects (FAO)	MCAD deficiency, VLCAD deficiency, CPT II deficiency
Serum insulin & C‑peptide	Inappropriate insulin secretion during hypoglycemia	Congenital hyperinsulinism, insulinoma
β‑hydroxybutyrate	Distinguishes between ketotic and nonketotic hypoglycemia	Low ketones: hyperinsulinism; FAO disorders; high ketones: ketotic hypoglycemia
Growth hormone	Growth hormone deficiency	Congenital GH deficiency, hypopituitarism
Cortisol	Adrenal insufficiency	Primary adrenal insufficiency (Addison's disease)
Serum lactate	Mitochondrial dysfunction or disorders of energy metabolism	Mitochondrial disorders, pyruvate dehydrogenase deficiency
Uric acid	Purine metabolism disorders	Lesch‑Nyhan syndrome, PRPP synthetase overactivity
Lipid panel	Metabolic dyslipidemias and FAO disorders	Familial hyperlipidemia, FAO defects
Ammonia	Urea cycle disorders	Ornithine-trans-carbamylasedeficiency (OTC), citrullinemia
Pyruvate	Disorders of gluconeogenesis or mitochondrial metabolism	Pyruvate carboxylase deficiency, mitochondrial disorders
Urine ketones	Ketone production during hypoglycemia	Absent ketones: hyperinsulinism; elevated ketones: ketotic hypoglycemia

During her PICU stay, a supervised oral feeding trial was followed by emesis and a significant hypoglycemic episode, with a glucose nadir of 44 mg/dL despite ongoing intravenous dextrose at a GIR of 6.7 mg/kg/min, representing a critical diagnostic turning point in her course. This event prompted the collection of comprehensive critical samples at the time of hypoglycemia. Glucose levels were subsequently corrected, and the GIR was escalated to 8.4 mg/kg/min to maintain euglycemia. A peripherally inserted central catheter (PICC) line was then placed to ensure reliable vascular access for ongoing management and frequent monitoring.

**Figure 2:**
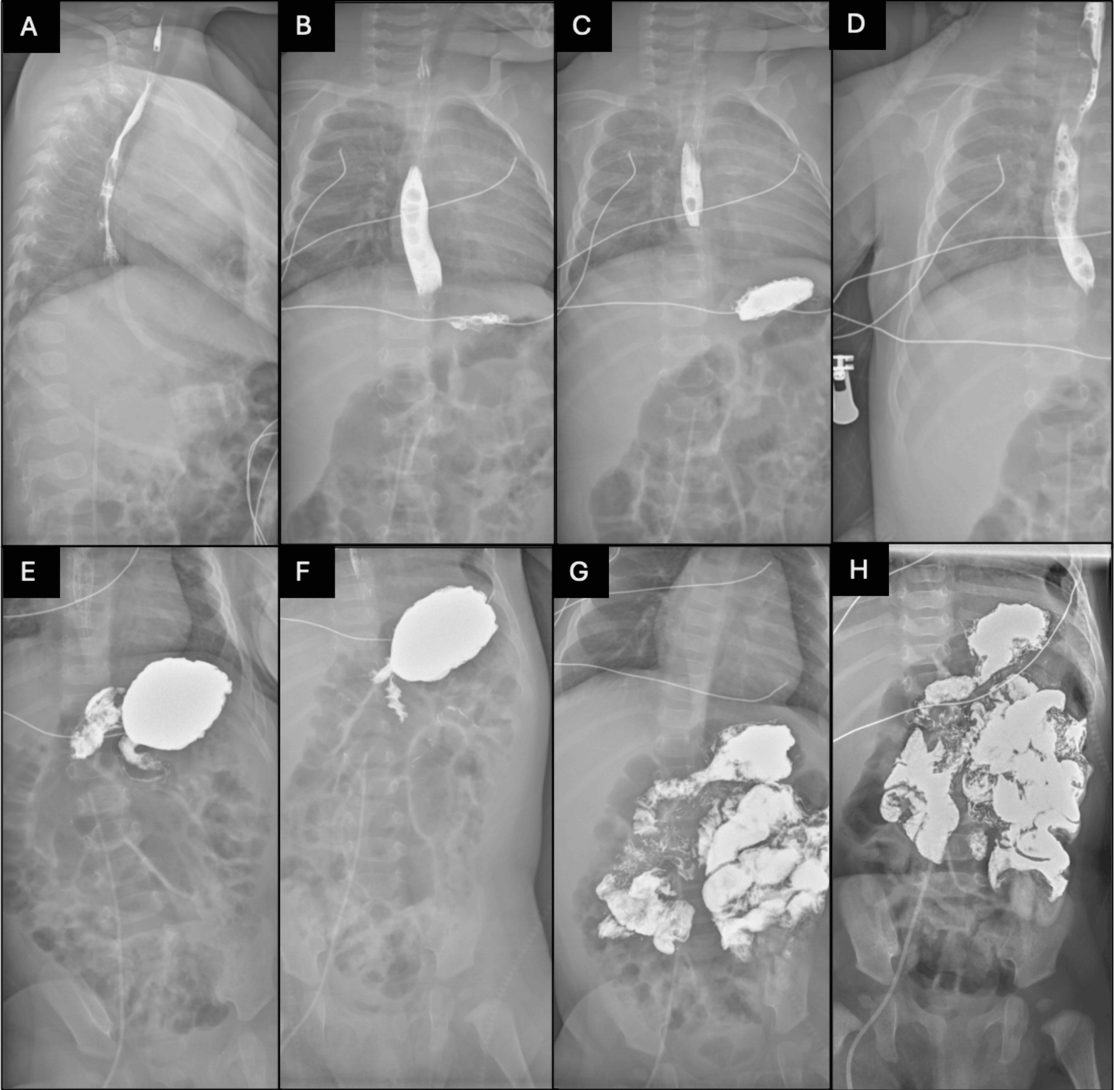
X-ray upper GI series after contrast ingestion with small bowel follow-through GI: gastrointestinal (A) Lateral view showing initial contrast passage through the esophagus. (B) Anteroposterior view demonstrating contrast within the mid-esophagus. (C) Continued esophageal transit with contrast entering the stomach. (D) Contrast outlining the distal esophagus without evidence of obstruction. (E) Contrast within the stomach with the normal configuration. (F) Gastric emptying with passage of contrast into the duodenum. (G) Contrast progression through proximal small bowel loops. (H) Delayed image demonstrating normal distribution of contrast throughout the small intestine without evidence of malrotation or obstruction

Given the recurrent vomiting, an upper gastrointestinal series with small bowel follow-through was performed and was reportedly unremarkable. The hypoglycemia panel demonstrated detectable insulin and C-peptide levels despite low plasma glucose. Although the absolute insulin value appeared numerically low, insulin should be fully suppressed during significant hypoglycemia; therefore, any measurable insulin in this context is considered inappropriately elevated, consistent with dysregulated insulin secretion. Cortisol and growth hormone were within appropriate parameters, and urine ketones were in trace amounts. Follow-up metabolic studies demonstrated improvement in metabolic acidosis with normalization of serum bicarbonate, downtrending transaminases, and recovery of the absolute neutrophil count. Figure 2 shows an X-ray upper GI series after contrast ingestion, followed by a small bowel follow-through of the contrast.

A nasoduodenal tube was placed, and enteral feeding was initiated. However, with the advancement of feeds, the patient developed recurrent hypoglycemia, despite escalation of the GIR to 13.3 mg/kg/min. Intravenous dextrose boluses were administered for correction, but the hypoglycemia paradoxically worsened following these boluses, raising further concern for an insulin-mediated process. Given the strong clinical suspicion for hyperinsulinemic hypoglycemia (HH) and the anticipated delay in results of the critical metabolic profile, a bedside glucagon stimulation test was performed. This demonstrated a marked rise in plasma glucose from 40 mg/dL to 131 mg/dL within 20 minutes of glucagon administration, strongly supporting the diagnosis of hyperinsulinism. 

Endocrinology recommended sending a genetic hypoglycemia panel and initiating therapy with diazoxide in combination with hydrochlorothiazide to mitigate the side effect of fluid retention. The patient demonstrated an excellent clinical response, with gradual tapering of the GIR and successful progression to full oral feeds. As blood glucose levels remained stable, intravenous fluids were discontinued, and she was transitioned from the PICU to the general pediatric ward. 

Meanwhile, the metabolic workup revealed suppressed β-hydroxybutyrate (0.9 mmol/L) with normal free fatty acid levels, findings that further supported hyperinsulinism, as ketone bodies, particularly β-hydroxybutyrate, and free fatty acids typically increase during hypoglycemia to provide alternative energy substrates. Additional metabolic studies, including plasma amino acids, acylcarnitine profile, and urinary organic acids, were within normal limits. Genetic testing with a CHI panel did not identify any pathogenic variants. The critical Lab investigation results are shown in Table 5. 

**Table 5:** Critical laboratory evaluation during hypoglycemia GH: growth hormone

Test/panel	Result	Normal reference (infant)	Interpretation of our patient
Plasma amino acids	Normal	Normal pattern for age	No evidence of aminoacidopathy
Urinary organic acids	Normal	Normal pattern for age	Organic acidemias unlikely
Acylcarnitine profile	Normal	Normal pattern for age	Fatty acid oxidation disorders unlikely
Serum insulin	1 μU/mL	<2 μU/mL (typically suppressed during hypoglycemia)	Inappropriately detectable during hypoglycemia, suggesting insulin-mediated process
C‑peptide	1.20 ng/mL	1.10-1.40 ng/ml (suppressed during hypoglycemia)	Confirms endogenous insulin secretion
β‑hydroxybutyrate	0.90 mmol/L	>1.80 mmol/L during hypoglycemia	Inappropriately suppressed ketone production, consistent with hyperinsulinism
Growth hormone	1.90 ng/mL	>10 ng/mL during hypoglycemia	Low level but not diagnostic; A single low GH value during acute illness is nonspecific; in this context of clear biochemical hyperinsulinism, GH deficiency is unlikely to be the primary driver of hypoglycemia
Cortisol	24.20 µg/dL	>18 µg/dL during hypoglycemia	Appropriate stress response; adrenal insufficiency unlikely
Serum lactate	1.10 mmol/L	0.50-2.20 mmol/L	No evidence of mitochondrial or severe metabolic disorder
Uric acid	4.60 mg/dL	2-6 mg/dL	Within normal limits
Lipid panel	Normal	Age‑appropriate normal range	Metabolic dyslipidemia unlikely
Ammonia	<10 µmol/L	<50 µmol/L	Urea cycle disorders unlikely
Pyruvate	0.6 mmol/L	0.3-0.7 mmol/L	Gluconeogenesis disorders unlikely
Urine ketones	10 mg/dL	Positive during fasting	Low ketone response relative to the degree of hypoglycemia, supporting hyperinsulinism

**Figure 3:**
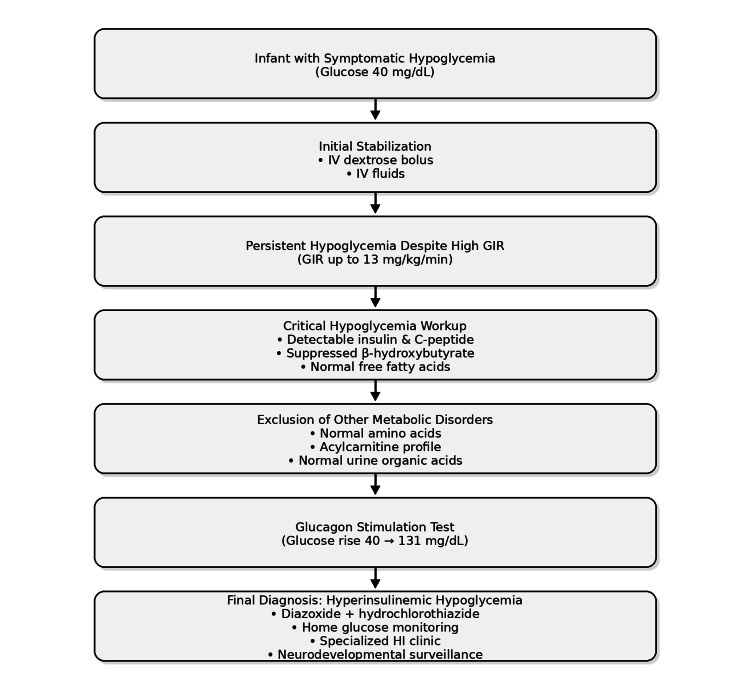
Clinical and biochemical pathway leading to the diagnosis of hyperinsulinemic hypoglycemia

After a 24-hour observation period with consistently normal glucose values, the patient was discharged home on diazoxide and hydrochlorothiazide therapy. She was provided with a home glucometer and instructed to check blood glucose every three hours, with a clear emergency plan for hypoglycemia management. Follow-up in the endocrinology clinic within one week of discharge allowed for adjustment of diazoxide dosing, and continuous glucose monitoring was initiated for ongoing surveillance. She was also established with a specialized hyperinsulinism clinic for continued management and treatment. Given the risk of hypoglycemia-related neurological injury, the patient will also undergo longitudinal neurodevelopmental surveillance as part of her ongoing care. Figure 3 shows a summary of events leading to the final diagnosis of HH.

## Discussion

BRUE are uncommon yet clinically consequential presentations in infancy that necessitate careful risk stratification into lower and higher risk categories to guide evaluation and identify potentially serious underlying pathology. Lower-risk BRUE is defined by the AAP on the basis of several reassuring features: age >60 days, gestational age at birth ≥32 weeks with a postconceptional age ≥45 weeks, a first and isolated event lasting <1 minute, no requirement for cardiopulmonary resuscitation by a trained medical provider, and the absence of concerning historical features or abnormal findings on physical examination. Infants who do not meet these criteria are considered higher risk and warrant a more comprehensive diagnostic assessment [1].

A recent systematic review and meta-analysis demonstrated a clear correlation between BRUE recurrence and the presence of serious underlying diagnoses. While overall mortality was rare, approximately 6% of infants were ultimately found to have a significant medical condition, and 13.6% experienced recurrent events within three months, indicating that repetition of BRUE markedly increases the probability of an occult pathology [4]. This association was evident in our case, where a significant family history of sudden sibling deaths prompted close clinical surveillance. The patient experienced two prior BRUE episodes; however, continued monitoring enabled timely reassessment, and the clue of hypoglycemia ultimately led to the diagnosis of CHI. 

This case also highlights a potential missed opportunity for earlier diagnosis. In the context of a significant family history, the infant met criteria for a higher-risk BRUE, warranting a more comprehensive, tiered evaluation that includes baseline laboratory studies [5]. Although the initial admission appropriately focused on potential cardiac etiologies, neither glucose monitoring nor basic laboratory testing was performed; inclusion of these assessments may have allowed earlier identification of hypoglycemia and expedited diagnosis. Similarly, a subsequent emergency department presentation with seizure-like activity, possibly reflecting hypoglycemia, resulted in discharge after only six hours of observation without a more thorough metabolic evaluation. Collectively, these events emphasize the need for individualized risk assessment and a heightened index of suspicion beyond standard BRUE stratification, particularly in the setting of recurrent or atypical presentations.

Maintenance of normoglycemia requires a dynamic balance between exogenous glucose supply and endogenous energy reserves, such as glycogen, fatty acids, and gluconeogenic substrates such as amino acids, glycerol, and lactate. This process depends on an intact endocrine network that coordinates fuel mobilization and utilization through tightly regulated pathways of glycogenesis, glycogenolysis, glycolysis, gluconeogenesis, lipolysis, and ketogenesis [6]. 

In HH, excess insulin drives rapid glucose uptake into the skeletal muscle, adipose tissue, and liver, producing profound hypoglycemia. Concurrent suppression of glycogenolysis, gluconeogenesis, lipolysis, and ketogenesis results in a hypoketotic state, depriving the brain of alternative fuels. Neonates and infants are particularly vulnerable due to their high cerebral glucose demand during a critical period of neurodevelopment [7,8]. 

Since hypoglycemia was the initial diagnostic indicator in our case, it is always essential to collect critical samples during the hypoglycemic episode for further evaluation of etiology. Blood glucose levels, ketones, insulin and C-peptide, cortisol, growth hormone, and lactate should be prioritized for testing before treatment, along with the first urine sample for organic and amino acids, reducing substances, glucose, and ketones. Additional tests can be considered after treatment, such as liver function tests, ammonia, free fatty acids, amino acids, and acylcarnitine [9].

When HH is suspected, key diagnostic features include a plasma glucose level <54 mg/dL in the presence of detectable or inappropriately normal insulin and C-peptide, accompanied by suppressed β-hydroxybutyrate and free fatty acids, and an increased glucose infusion requirement (>8 mg/kg/min) [6]. In our patient, these criteria were clearly met: a plasma glucose of 44 mg/dL was associated with measurable insulin and C-peptide levels, along with suppressed β-hydroxybutyrate and absence of ketonuria, while glucose requirements escalated to a GIR of 13.3 mg/kg/min to maintain euglycemia.

The glycemic response to glucagon provides an estimate of liver glycogen levels. The glucagon stimulation test played a crucial role in diagnosing our case while waiting for genetic and metabolic evaluations. In theory, a positive response is shown by an increase in glucose of ≥30 mg/dL following glucagon treatment, which indicates inappropriate insulin activity despite having sufficient hepatic glycogen stores and acts as a reliable tool to distinguish hyperinsulinism from other causes of hypoglycemia [9]. 

The cornerstone of management is early recognition and prompt therapy. Acute stabilization involves 2 mL/kg of 10% dextrose intravenously, but repeated boluses should be avoided as they may stimulate further insulin release [6]. This phenomenon was observed in our patient, where recurrent boluses precipitated worsening hypoglycemia until definitive stabilization was achieved with diazoxide. 

Diazoxide is the first-line pharmacologic therapy and acts via the SUR1 subunit of the ATP-sensitive potassium (KATP) channel; therefore, efficacy depends on channel integrity [6]. Clinical response is evidenced by a decline in glucose infusion requirements toward physiologic levels [10]. Its use may be limited by fluid retention, cardiac dysfunction, electrolyte imbalance, and pulmonary hypertension; co-administration of a thiazide diuretic to mitigate the adverse effects is routinely employed [6]. For diazoxide-unresponsive cases, options include octreotide or long-acting somatostatin analogs, and in selected refractory patients, sirolimus, with close monitoring for adverse effects [11]. 

CHI is genetically heterogeneous, involving mutations in multiple genes regulating β-cell secretion, including ABCC8, KCNJ11, GLUD1, GCK, HADH, SLC16A1, UCP2, HNF4A, HNF1A, HK1, KCNQ1, CACNA1D, FOXA2, EIF2S3, PGM1, and PMM2 [12]. A landmark cohort from the Children’s Hospital of Philadelphia demonstrated that recessive KATP mutations predict diffuse, diazoxide-unresponsive disease, whereas mutations such as GLUD1, HADH, HNF4A, and HNF1A are more often medically responsive, emphasizing the value of molecular diagnosis in guiding individualized therapy and surgical referral [13]. 

Another important observation from the study is that more than half of diazoxide-responsive patients lacked identifiable genetic mutations, suggesting the presence of yet-undiscovered loci [13]. This aligns with our patient, who demonstrated an excellent clinical response despite a negative CHI gene panel. Such findings are consistent with reported cohorts in which a substantial proportion of clinically and biochemically confirmed CHI cases have no detectable pathogenic variants, illustrating that the diagnosis remains fundamentally clinical and biochemical rather than solely genotype-driven.

## Conclusions

Persistent hypoglycemia requiring high GIRs, particularly in the setting of hypoketosis, should prompt early evaluation for CHI, especially in infants presenting with recurrent BRUE-like events. Timely recognition of this treatable endocrine emergency is critical not only for prompt metabolic stabilization but also for safeguarding long-term neurodevelopmental outcomes. In such contexts, the glucagon stimulation test serves as a rapid and practical bedside diagnostic tool while definitive studies are pending. This case illustrates how delayed consideration of hypoglycemia may represent a missed opportunity for earlier diagnosis and intervention, highlighting the need for early glucose assessment and a sustained index of suspicion in recurrent or atypical presentations, even when initial evaluations appear reassuring.
